# Aortic Valve Repair for Severe Commissural Leaflet Defects Using Aortic Wall Patches

**DOI:** 10.1016/j.atssr.2025.01.020

**Published:** 2025-02-21

**Authors:** Alexander P. Nissen, Melissa M. Levack, Vinay Badhwar, W. Brent Keeling, J. Scott Rankin

**Affiliations:** 1 Division of Cardiothoracic Surgery, Emory University School of Medicine, Atlanta, Georgia; 2 Division of Cardiothoracic Surgery, Centura Health, Denver, Colorado; 3 Department of Cardiovascular and Thoracic Surgery, West Virginia University, Morgantown West Virginia

## Abstract

**Background:**

In patients with aortic insufficiency, annular dilatation often accompanies valve incompetence, necessitating annuloplasty. However, primary leaflet defects also are common, and when found unexpectedly at the time of planned repair, inadequate leaflet tissue often prompts prosthetic valve replacement. A method for achieving stable repair for severe leaflet deficiencies would be useful.

**Methods:**

In this report, major leaflet defects due to ruptured large fenestrations were encountered in 2 patients, the first repaired with extensive plication, which failed. In the second patient, the defect was reconstructed using an autologous aortic wall patch. After geometric annuloplasty, the aortic wall strip was sutured with interrupted 6-0 sutures from the nodulus to the commissural top, with the intima facing coaptation. Leaflet free-edge length was adjusted to match the other normal leaflets at approximately reconstructed annular diameter x 1.5.

**Results:**

In the aortic wall patch patient, grade 4 preoperative aortic insufficiency fell to zero after repair, and the patient is doing well with continued excellent echo parameters at 1 year postoperatively.

**Conclusions:**

As a leaflet substitute during aortic valve repair, aortic wall patches seem to provide an excellent solution to managing severe leaflet deficiencies.

**In Short**

▪ When deficient leaflet tissue is encountered during aortic valve repair, patches of autologous ascending aortic wall seem to provide an excellent leaflet substitute.
▪ Techniques for insertion are simple, and results are good to the intermediate term.

In cases of chronic aortic insufficiency (AI), valve incompetence almost always is accompanied by significant enlargement of annular diameter, averaging 4.9 mm of dilatation in one series (Figure 1).^1^ Thus, most would agree that annuloplasty is an important component of aortic valve repair for AI. Moreover, when considering patients with grade 3-4 AI, it is somewhat surprising that 80% also exhibit notable leaflet defects.^2^ Most defects can be repaired readily with free-edge plication for prolapse or nodular release for nodular retraction.^3^, ^4^, ^5^ When severe leaflet deficiencies are encountered, however, such as large ruptured fenestrations, valve replacement frequently is required. While leaflet replacement with pericardium has been used, pericardial degeneration limits outcomes.^6^ In fact, a recent study from the German Aortic Root Repair Registry of 762 aortic valve repair patients with fenestrations^7^ indicated that 12% underwent immediate prosthetic valve replacement; when repaired, fenestrations in more than one cusp, or large leaflet inhomogeneities, were associated with inferior outcomes (freedom from major adverse cardiovascular events of only 41% at 3 years). Thus, a method for repairing valves with more severe leaflet defects would be useful, especially in nonelderly patients, in order to realize the long-term benefits of native tissue preservation. In a recent series, major leaflet and commissural defects were reconstructed using living autologous aortic wall patches,^8^ with good results approaching 3 years of maximal follow-up. This approach also was extended to full leaflet replacement with good outcomes.^9^ The current report and video illustrate the application of aortic wall patches to reconstruct large ruptured leaflet fenestrations.

**Figure 1:**
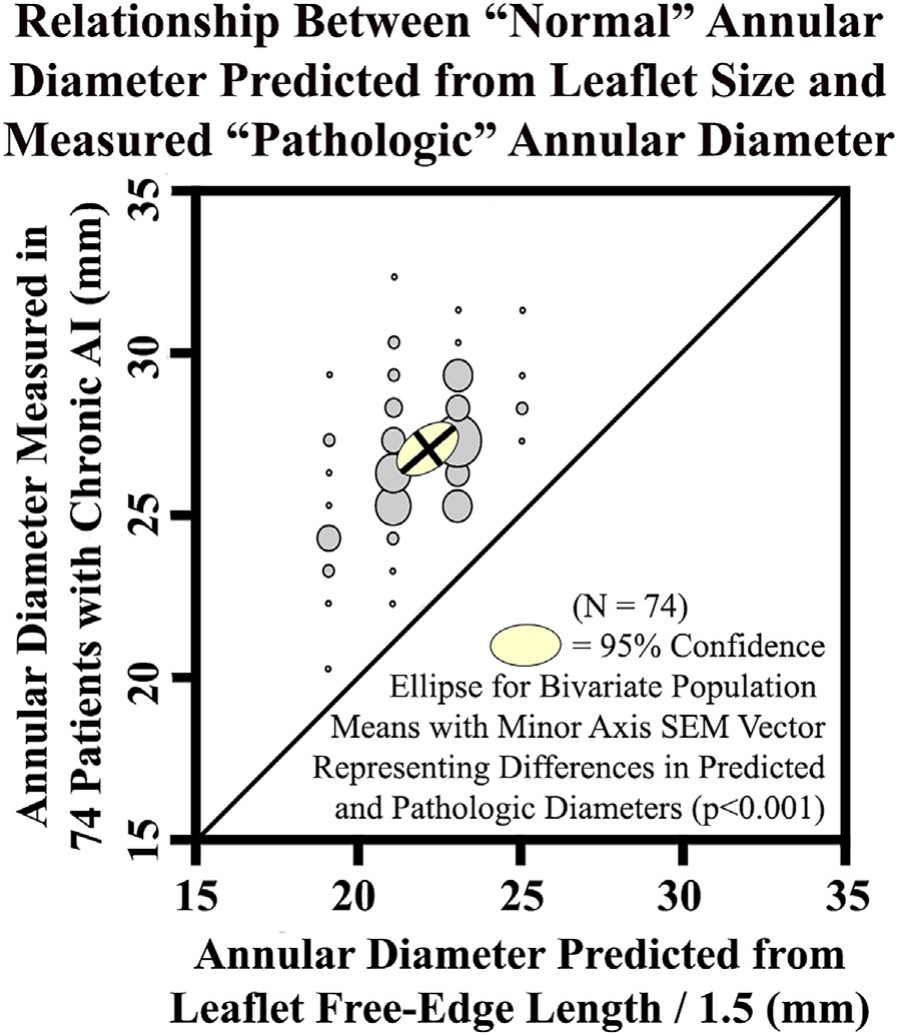
Comparison of “normal” annular diameter predicted from leaflet free-edge length and pathologic annular diameter assessed with Hegar dilators in 74 patients with chronic aortic insufficiency (AI). Measured annular diameter was enlarged to some extent in every patient. The 95% covariate confidence ellipse of mean values did not intersect the line of identity, indicating significant annular dilatation (minor axis standard error of the mean [SEM] vector) in most patients with chronic AI. Average pathologic dilatation (y-axis of ellipse center vs line of identity) was 4.9 ± 2.1 mm. Modified from Jasinski and associates.^1^

## Patients and Methods

In the Video, 2 techniques for reconstruction of ruptured large fenestrations are illustrated, the first being an older method^2^ involving extensive leaflet plication. Because of suboptimal results with the first approach, the second patient with a ruptured fenestration had the lateral leaflet reconstructed with a patch of autologous aortic wall.

## Results

The first patient was a 60 year-old man with near sudden death and severe AI causing moderate left ventricular dysfunction. The echocardiogram showed a severe posterior AI jet, similar to isolated right leaflet prolapse—but with a flail leaflet target, consistent with commissural rupture. The annulus was 28 mm, and all 3 leaflets sized to a 21-mm annuloplasty ring. The ring posts and body were sutured to the aortic annulus, and both the left and non-coronary leaflets looked good. But the right leaflet was severely prolapsing due to a large ruptured fenestration. The right leaflet was plicated to correct the prolapse. Because of a large tissue defect, the reconstruction was difficult and required 6 plications. The leaflets coapted well, although concerns existed about inadequate leaflet in the commissure. Post repair, the valve was competent with a mean gradient of 12 mm Hg, and the patient did well—until 1 year after repair—when the right leaflet ruptured again, producing recurrent posterior AI. At reoperation, the left and noncoronary leaflets still looked good, but the repaired right leaflet had torn again at the commissure with severe recurrent prolapse. The leaflets were excised, and it was reassuring to see how nicely the ring had healed to the inflow aspect of the annulus with a thin fibrous capsule and good endothelialization. The valve was replaced, and the patient did well.

**Figure 2:**
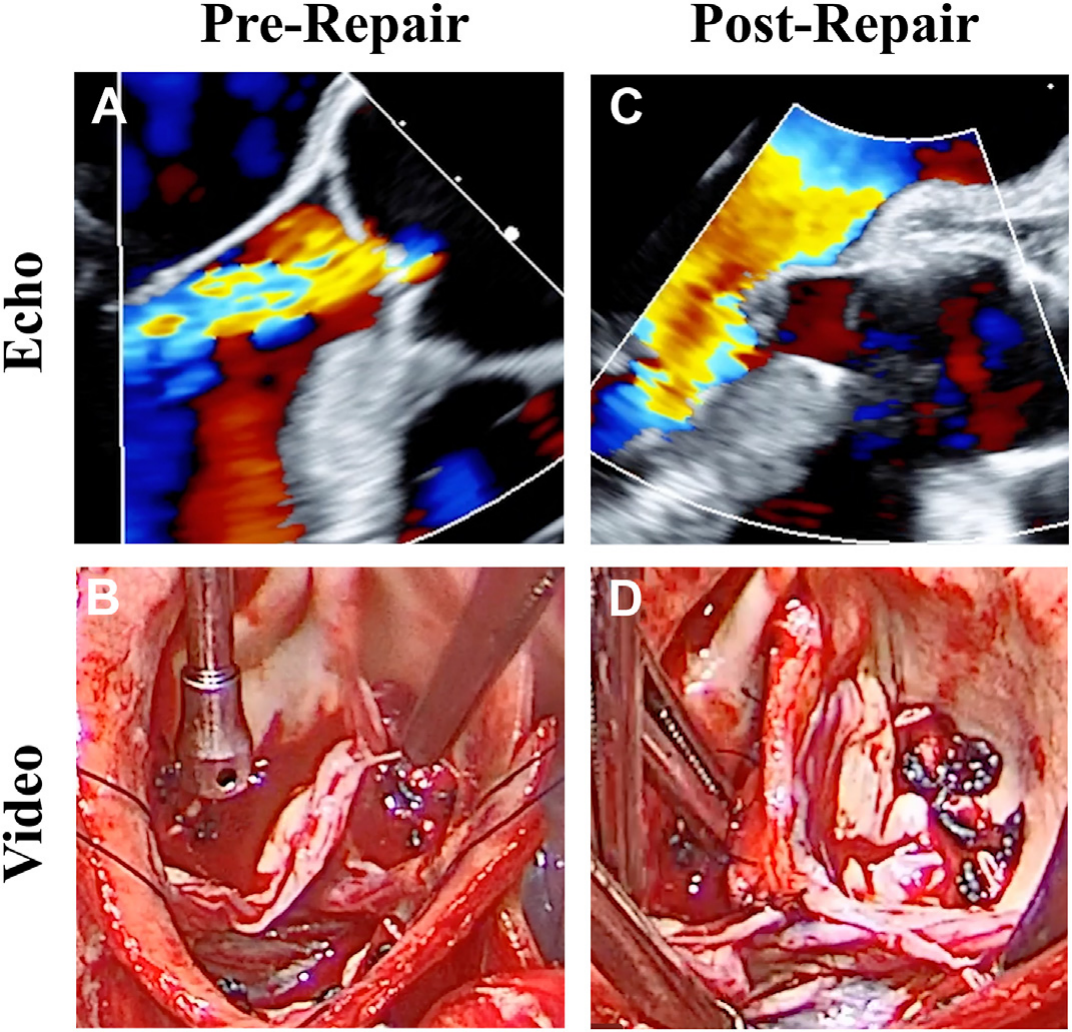
Echocardiographic and video views of valve for patient 2 showing severe posterior aortic insufficiency (A) from ruptured right coronary fenestration (B). After repair, valve was fully competent (C) with strip of aortic wall sutured into the right leaflet and commissure (D).

The second patient was a 61-year-old man with a 6-cm ascending aortic aneurysm involving the arch, also with severe AI and reduced left ventricular function. As with patient 1, the AI jet was posterior, with a mobile target suggestive of commissural rupture (Video). The annulus was 24 mm, and the root was normal size. The leaflets sized to a 23-mm geometric ring, which was sutured to the 3 subcommissural triangles and sinus annular aspects with interrupted sutures. The ruptured right leaflet fenestration was primarily causing the prolapse, but after the central leaflet was plicated, insufficient leaflet existed in the commissure. A strip of ascending aortic wall was harvested from the distal aortotomy and sutured to the nodulus. The patch then was sutured progressively to the leaflet free-edge using interrupted 6-0 Prolene (Ethicon) sutures (Video). The patch intima faced the coaptation, and the knots were on the sinus side. The top patch suture was placed as a pledgetted mattress suture to the outside of the aorta. Excess patch was excised leaving the strip 2 mm taller than the other leaflets. An additional suture was placed centrally to the leaflet free-edge to make the patch stand more vertically and to achieve free-edge lengths for all 3 leaflets of approximately annular diameter (23 mm) x π/2 (or 36 mm).^4^ Ultimately the autologous aortic patch created a tall right leaflet with good central coaptation. On echocardiogram, the valve was fully competent, with good opening of the patch, adequate leaflet position (Figure 2), and a mean gradient of 11 mm Hg. At 1 year of follow-up, his clinical status and echo findings were excellent, with improved left ventricular function.

## Comment

Although AI frequently occurs in the setting of annular and/or proximal aortic dilatation, concomitant leaflet defects requiring reconstruction also are present in up to 80% of patients with moderate-to-severe AI.^2^ That number seems high when reflecting on reimplantation publications for aneurysms, but many of these series have substantial patient proportions with none-to-moderate AI. While the most common leaflet derangement, prolapse, is now readily managed with central leaflet plication, other less-common defects with deficient leaflet tissue are still problematic. A difficult group has been patients with large ruptured fenestrations and deficient leaflet area.^6^^,^^7^ Attempting to repair such defects with only plication leaves the leaflet under inordinate stress and is prone to late failure, as in patient 1. Adding tissue to such deficient leaflets is an obvious solution, but pericardial patch repairs have been independently associated with recurrent AI because of late pericardial degeneration. For this reason, a series of patients having no other repair option was selected, and after detailed informed consent, underwent autologous aortic wall patches as leaflet substitutes.^8^ The rationale for this approach is that the patient’s ascending aorta is a living autologous vascular tissue that derives its nutrition from luminal diffusion, is accustomed to systemic pressure, can be routinely harvested at the time of aortic valve repair, and requires simple operative methods. The earliest patients managed with this approach are now almost at 3 years postoperative, and late echocardiograms appear excellent and similar to those obtained during the operative repair. Formal late follow-up studies are planned, but outcomes at present seem quite satisfactory.

In addition to augmenting leaflet area, this technique extends the zone of coaptation, and the repaired valve may tolerate mild reconstruction inadequacies over time without a diminished coaptation height or recurrent AI. The living aortic tissue should not contract or calcify, and is strong enough to maintain its initial geometry. Specifically, the commissural regions are under the highest stress and can be the most challenging to repair. The high-stress commissural aspect of the leaflet is thin, offers the least tissue redundancy, and is difficult to work with in terms of leaflet suturing or plication. Adding a strong aortic wall patch to the commissure not only overcomes each of these problems, but also increases commissural strength by carrying the final commissural sutures to the outside of the aorta. With these factors in mind, reconstruction of large commissural ruptures using patches of autologous aortic wall holds promise as a simple and reproducible technique for achieving stable long-term competence.
